# Multiple Self-Supervised Auxiliary Tasks for Target-Driven Visual Navigation Using Deep Reinforcement Learning

**DOI:** 10.3390/e25071007

**Published:** 2023-06-30

**Authors:** Wenzhi Zhang, Li He, Hongwei Wang, Liang Yuan, Wendong Xiao

**Affiliations:** 1 School of Mechanical Engineering, Xinjiang University, Urumqi 830046, China; 107552103919@stu.xju.edu.cn (W.Z.);; 2 School of Information Science and Technology, Beijing University of Chemical Technology, Beijing 100029, China

**Keywords:** target-driven visual navigation, deep reinforcement learning, self-supervised auxiliary tasks, representation learning

## Abstract

Visual navigation based on deep reinforcement learning requires a large amount of interaction with the environment, and due to the reward sparsity, it requires a large amount of training time and computational resources. In this paper, we focus on sample efficiency and navigation performance and propose a framework for visual navigation based on multiple self-supervised auxiliary tasks. Specifically, we present an LSTM-based dynamics model and an attention-based image-reconstruction model as auxiliary tasks. These self-supervised auxiliary tasks enable agents to learn navigation strategies directly from the original high-dimensional images without relying on ResNet features by constructing latent representation learning. Experimental results show that without manually designed features and prior demonstrations, our method significantly improves the training efficiency and outperforms the baseline algorithms on the simulator and real-world image datasets.

## 1. Introduction

Autonomous navigation is a prerequisite for mobile robots to accomplish complex tasks. Visual navigation is receiving increasing attention due to its advantages of rich information and low cost. Conventional visual-navigation systems incorporate modules for simultaneous localization and mapping (SLAM) [1,2], path planning [3], and motion control. However, integrating these modules can accumulate computational errors, and navigation quality depends on high-cost maps that are challenging to adapt to complex, unstructured environments [4]. In recent years, the remarkable success of deep reinforcement learning (DRL) algorithms in domains such as Go [5,6] and Atari games [7] has sparked research interest in DRL-based visual navigation methods for mobile robots [8]. The availability of indoor photo-level simulators [9,10] and pre-trained datasets [11,12] has led to an explosion in DRL-based visual navigation research [13]. Evidence suggests that DRL-based visual navigation methods outperform traditional methods in complex, unstructured indoor environments [14]. However, DRL-based visual navigation has two critical unresolved issues: the training process could be more efficient and the navigation performance is mainly validated in simulators.

To enhance training efficiency and improve navigation performance, Du [15] proposed object relationship graphs capable of locating invisible targets within the current view. They also employed imitation learning to drive the training process, which provided expert experiences to the agents and prevented the navigation network from becoming stuck in a deadlock. These methods demonstrated a significant improvement in the success rate of visual navigation in unseen environments. In another study, Mayo [16] introduced a novel attention probability model that encoded semantic information about observed objects and spatial information about their locations. By combining semantic and spatial information, the model motivated the agent to navigate efficiently toward the desired objects. Furthermore, Xiao [17] integrated single-step reward observation and collision penalties into the classical A3C framework. They also introduced the Observation-Action Consistency (OAC) model to ensure that the agent reached subgoals and moved closer to the targets sequentially. However, the above methods utilize pre-trained features (e.g., Resnet18, Resnet50), which may not be beneficial for a specific visual navigation tasks. That is due to the disparity between the visual navigation environment where the agent is placed and the environment in which the pre-trained features were learned.

Kulhánek [18] employed the original high-dimensional images directly as input to the agent and developed the navigation-related representation learning through auxiliary tasks such as pixel control and reward prediction. The proposed method’s effectiveness in enhancing model training efficiency and the navigation success rate was validated in AI2THOR and House3D. To enable the training of navigation strategies using real-world image datasets, ref. [19] proposed image reconstruction tasks, eliminating the need for manual labeling of images. Building upon this work, we introduce an LSTM-based dynamics model and an attention-based image reconstruction model as auxiliary learning tasks. These tasks are jointly trained alongside pixel control, depth map prediction, and reward prediction. Our method significantly improves the model’s training efficiency and navigation performance. The main contributions of this article are as follows:

(1) A general DRL framework for target-driven visual navigation is established, in which multiple self-supervised auxiliary tasks can be integrated easily.
(2) Use the LSTM-based dynamics model to establish effective representation learning for navigation tasks, which significantly improves the learning efficiency of DRL.
(3) An attention-based image reconstruction model is designed to pay more attention to the critical regions in the navigation task, making it easier for the navigation agents to understand the scene and make decisions more accurately.
(4) The effectiveness of our method was validated in a simulator. However, due to the closer resemblance of real-world image datasets to the actual environment, we further validated the performance of our method on real-world image datasets.

The remaining sections of the paper are organized as follows: Section 2 reviews related work, Section 3 provides background knowledge on reinforcement learning, Section 4 presents the proposed approach, Section 5 showcases experimental results, and finally, Section 6 concludes the paper.

## 2. Related Work

DRL-based visual navigation has received significant attention in recent years. Oh [20] utilized DRL to complete a maze-walking game and introduced a DRL architecture capable of storing recently observed images to construct memory queries based on temporal context. Experimental results demonstrated that this structure improved the generalization ability. Zhu [8] proposed a target-driven visual navigation network that utilized both observed and target images as inputs. This approach enabled the neural network to process new targets. Zeng [21] introduced an asynchronous proximal policy optimization algorithm to reduce training variance during the agent’s interactions with the environment and ensure the monotonic improvement of the visual navigation strategy. Devo [22] extended the target-driven visual navigation network by incorporating a target localization network ahead of the navigation network. This addition allowed the system to identify specific targets within the robot’s field of view. Wu [23] focused on the semantic visual navigation problem and proposed a Bayesian Relational Memory architecture to enhance semantic visual navigation intelligence in unseen environments. Chang [24] trained an agent to navigate to objects of interest in a new environment by learning and utilizing semantic cues from videos, specifically by observing YouTube videos. Vision-and-Language Navigation is a more complex navigation task, where the agents need to follow natural language instructions and navigate in a visual environment. Refs. [25,26,27] combined the advantages of deep reinforcement learning and imitation learning to solve the visual-language navigation problem and achieved efficient navigation performance in datasets such as Room-to-Room (R2R) [28] and REVERIE [29]. Overall, these studies have contributed to the advancement of DRL-based visual navigation methods in various domains and have explored different techniques to enhance navigation performance.

Visual auxiliary tasks have been explored in visual navigation. Jaderberg [30] extended the asynchronous advantage actor-critic algorithm (A3C) by incorporating pixel control and reward prediction auxiliary tasks. Experimental results demonstrated that these auxiliary tasks significantly improved data efficiency. Similarly, ref. [31] extended the A3C algorithm by incorporating depth prediction and loop-closure-classification auxiliary tasks. This approach aimed to construct navigation-related representation learning and enhance training efficiency. Goel [32] utilized an optical flow-based approach to building a video motion model for objects in sequences. This model was employed for target segmentation, enhancing the agent’s ability to detect moving objects and reducing the need for extensive interactions with the environment. Agrawal [33] proposed a method to learn visual representations through motion, leveraging motion information to improve the learning of visual representations. This approach proved beneficial for perception tasks in unknown environments. Tongloy [34] integrated depth prediction and robot position estimation as auxiliary tasks for mobile robot navigation. Ye [35] employed inverse dynamics, temporal differencing, and action condition differencing as auxiliary tasks in PointGoal navigation tasks. The attention mechanism was used to aggregate the representation learning of each auxiliary task. Again, ref. [36] addressed the ObjectGoal navigation problem by incorporating auxiliary tasks such as the semantic segmentation of visual inputs and semantic target presence features. These studies highlight the effectiveness of visual auxiliary tasks in enhancing visual navigation performance and improving training efficiency.

The proposed approach differs from previous works in several key aspects. This paper focuses on the agent’s direct learning of navigation strategies from visual inputs without relying on ResNet features and semantic information. This paper also employs a series of self-supervised auxiliary tasks, which do not require labeled images and can be trained directly using real-world images. Additionally, instead of using the inverse dynamics model like [35], this paper designs an LSTM-based dynamics model. The LSTM network helps the dynamics model capture complex patterns and structures in the input sequence, providing a more robust feature representation for the navigation task. Ref. [26] uses an LSTM-based approach that combines object and room information to improve the performance of sequential BERT. Our proposed LSTM-based dynamics model uses the temporal-sequence modeling capability of LSTM to make the dynamics model more accurate in predicting the action and the next state. This paper utilizes self-supervised attention to reconstruct the target image from the observed image. That allows the generated scene graphs to be more tailored to the needs of the navigation task, making it easier for the navigation agent to understand the scenes and make more accurate decisions.

## 3. Background

### 3.1. Reinforcement Learning

**Figure 1:**
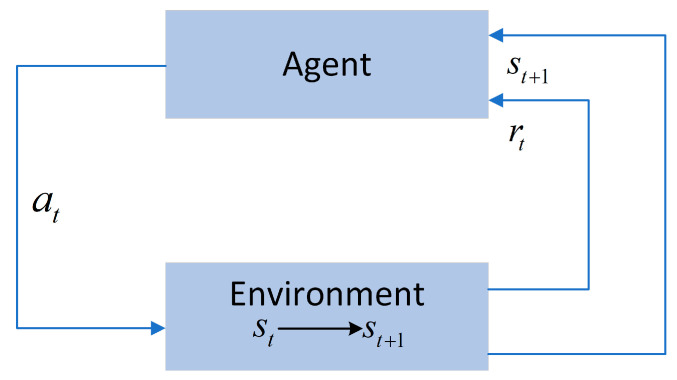
Reinforcement learning framework.

RL is a trial-and-error approach that relies on the continuous interaction between an agent and its environment. In visual navigation tasks, the goal of the navigation agent is to find the optimal set of actions from the current location to the target image. The agent receives the observed image and the target image as inputs. Suppose that at time step *t*, the state of the environment is denoted as $s_{t}$. Then, the agent takes the action $a_{t}$, the state of the environment transitions to $s_{t+1}$, and the agent receives a reward $r_{t}$. This is shown in Figure 1:

Continuously performing the above process, the agent attempts to learn action strategies by continuously interacting with a given environment, and the optimization goal of RL agent can be formally described as an objective function that maximizes the expected cumulative reward, as in Equations (1) and (2):

(1) $$G_{t}=r_{t}+\gamma r_{t+1}+\gamma^{2}r_{t+2}+\cdots +\gamma^{T-t}r_{T}=\sum_{k=t}^{T}\gamma^{k-t}r_{k}$$

(2) $$\pi^{*}=\arg\underset{\pi }{\max}E\left[\sum_{k=t}^{T}\gamma^{k-t}r_{k}\right]$$

There are three primary categories of methods for optimizing optimal strategies $\pi^{*}$: value-based methods, policy-based methods, and actor-critic methods. Actor-critic methods leverage the strengths of the previous two approaches. The policy network is crucial in determining the action selection. Meanwhile, the critic network is employed to estimate the value function, addressing the high variance in strategy gradient methods and improving training stability.

The actor-critic algorithm, initially developed for decision problems involving continuous action spaces [37], consists of a policy $\pi \left(a_{t}|s_{t};\theta \right)$ and an action-value function. This study utilizes a random strategy, a probability distribution over a discrete set of actions. The policy network is parameterized by θ, while the value network is parameterized by $\theta_{v}$. The optimization of these parameters is updated using the gradient descent method, as in Equations (3) and (4):

(3) $$\theta^{\prime }=\nabla_{\theta }\log\pi \left(a_{t}|s_{t};\theta \right)A\left(s_{t},a_{t};\theta ,\theta_{v}\right)+\beta \nabla_{\theta }H\left(\pi \left(s_{t};\theta \right)\right)$$

(4) $$\theta_{v}{}^{\prime }=\nabla_{\theta_{v}}\left[{\left(Q\left(s_{t},a_{t};\theta ,\theta_{v}\right)-V\left(s_{t};\theta_{v}\right)\right)}^{2}\right]$$

The advantage function, denoted as $A\left(s_{t},a_{t};\theta ,\theta_{v}\right)$, is computed using the n-step return estimate, as utilized by [38]. The final form of the advantage function is determined as in Equation (5):

(5) $$A\left(s_{t},a_{t};\theta ,\theta_{v}\right)=\sum_{i=0}^{n-1}\gamma^{i}r_{t+i}+\gamma^{n}V\left(s_{t+n};\theta_{v}\right)-V\left(s_{t};\theta_{v}\right)$$

$H\left(\pi \left(s_{t};\theta \right)\right)$ is the entropy of the policy; cross-entropy encourages exploration by preventing premature convergence of suboptimal deterministic policies.

To fully leverage computational resources, parallel training is commonly employed in DRL-based methods. One popular algorithm for parallel training is A3C [38], which utilizes a parallel approach to train multiple independent agents simultaneously in different environments. These agents share the same network structure and parameters. Each agent uses its own experience to update the shared parameters asynchronously. Similarly, the parallel advantage actor-critic (PAAC) [39] introduces a parameter master to manage and update the global model parameters. Each process can access the latest parameters from the parameter master, avoiding conflicts and redundant updates among different processes. This approach offers a more flexible and efficient way of sharing model parameters. In this paper, we adopt the parallel training approach of the PAAC algorithm and incorporate off-policy critic updates. An off-policy critic update uses historical trajectory data generated from previous policies. It does not require interaction with the current policy when updating the critic, thus making better use of previous experience and improving data utilization.

### 3.2. State Space

This paper captures the observation and target images from a monocular camera in a first-person view. The inclusion of target images in the state space aims to enable the agent’s learning process by navigating between different targets and enhancing the generalization performance of the navigation system. Furthermore, this paper incorporates the last action and the last reward as part of the state information, which gives the agent complete information.

### 3.3. Action Space

In this paper, the action space for the agent is discrete and defined as follows: A = {move forward, move backward, turn left, turn right, terminate}. Unlike the approaches described in [40,41], which rely on simulator termination signals to stop the agent as it closes the navigation target, real-world image datasets lack such termination signals. Therefore, similar to the approach presented in [42], this paper introduces a terminate action that enables the agent to detect whether it has reached the target automatically. The five discrete actions correspond to the numbers 0–4, which are chosen by the agent according to the probability distribution output by the policy network. We use a constant step length (0.2 m) and turning angle (90 degrees).

### 3.4. Reward Function

In our approach, we give the agent a reward of 1 when it completes the navigation task, which encompasses two primary standards: (i) the agent reaches the target, or (ii) the agent stops using the termination action. If the agent stops using the termination action without reaching the target (for example, if it stops in front of a bookcase while the target is a chair), a negative reward of −0.1 is assigned. In all other cases, the reward is set to 0. The reward function is defined as:

(6) $$\mathrm{reward}=\left\{ \begin{array}{l} 1.0,\;\mathrm{if}\;\mathrm{success}\\ -0.1,\;\mathrm{elif}\;\mathrm{collide}\\ 0.0,\;\mathrm{otherwise} \end{array}\right.$$

## 4. Method

### 4.1. Multi-Auxiliary Tasks PAAC Algorithm Framework

**Figure 2:**
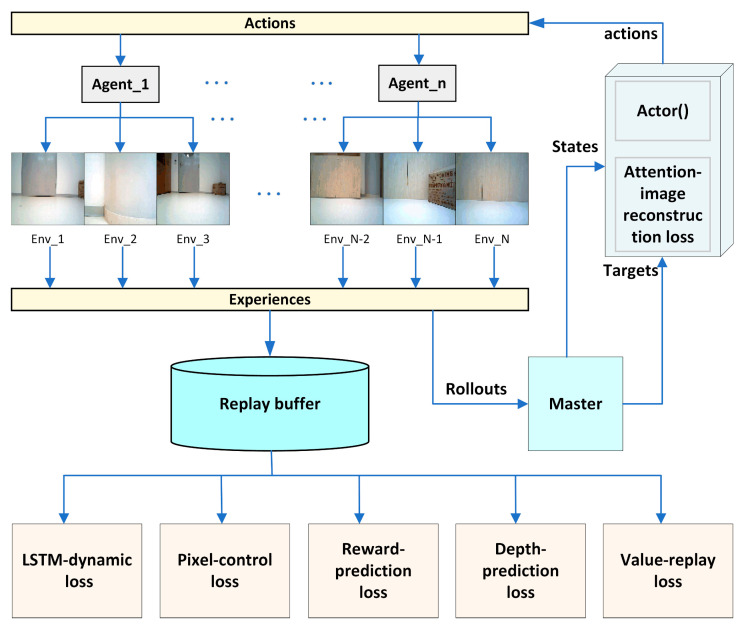
Proposed multi-auxiliary-task PAAC algorithm framework.

In this paper, the LSTM-based dynamics model, the attention-based image reconstruction model, the pixel-control model, the depth map prediction model, and the reward prediction model are jointly trained as auxiliary tasks with the navigation task. The overall framework of the model is shown in the following Figure 2.

The PAAC algorithm runs multiple agents in many parallel environments, each generating experience trajectories as it interacts with the environment. The experience trajectories are randomly sampled in the experience buffer and used to update the policy parameters. A replay buffer similar to DQN is used to improve data utilization. Data are sampled from the replay buffer to train the auxiliary tasks. In this paper, the LSTM-based dynamics model, pixel-control model, depth prediction model, reward prediction model, and value replay model are trained offline using data sampled from the replay buffer, and the attention-based image reconstruction model and policy network are trained online using data from the experience buffer. The self-supervised auxiliary tasks and the navigation task can be combined by sharing convolutional layers. Specifically, the first few convolutional layers of the feature-extraction module can be shared. By sharing the convolutional layers, the self-supervised auxiliary tasks can receive a richer representation of the environment and improve learning efficiency. Meanwhile, the navigation task can benefit from the additional supervised signals provided by the self-supervised auxiliary tasks to improve the model’s performance.

### 4.2. PAAC Algorithm Network Structure

**Figure 3:**
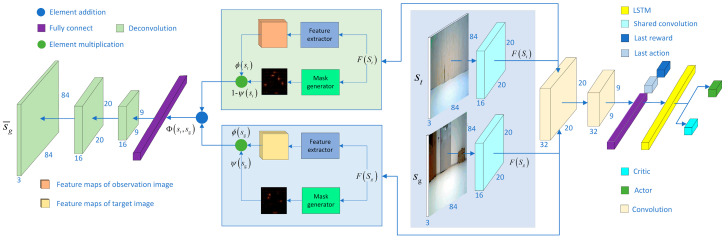
The PAAC algorithm and attention-based image reconstruction network structure.

The network input of the PAAC algorithm is the target image and the observed image, followed by the feature extraction module. The feature extraction module in this paper mainly uses a convolutional neural network. Each image enters a separate stream consisting of a convolutional layer with shared weight parameters. The output of the convolutional layers is stitched together and passed to two additional convolutional layers, followed by fully connected layers, each layer followed by an activation function. The features of the convolutional layers are spanned by the fully connected layers and merged with the last action and the last reward, fed to the LSTM layer, and finally to the actor and critic networks. Among them, the feature extraction module extracts the features of the input image through the convolutional layer, using shared weight parameters to enable the sharing of the parameters of the feature extraction process between different inputs to reduce the number of parameters and improve the training efficiency, as shown in Figure 3.

### 4.3. Attention-Based Image Reconstruction Network Structure

This paper reconstructs the target image from the observed image for the navigation task [43]. The specific process is as follows: the inputs are the observed image and the target image, and the features of the input image are extracted as $\phi \left(s_{t}\right)$ and $\phi \left(s_{g}\right)$, using the feature extraction module. Meanwhile, the mask maps of the observed image and the target image are output as $\psi \left(s_{t}\right)$ and $\psi \left(s_{g}\right)$, using the mask generator module to indicate the attention level to each pixel position. The features used to reconstruct the target image are calculated as Equation (7):

(7) $$\Phi \left(s_{t},s_{g}\right)=\left(1-\psi \left(s_{t}\right)\right)\cdot \left(1-\psi \left(s_{g}\right)\right)\cdot \phi \left(s_{t}\right)+\phi \left(s_{g}\right)\cdot \psi \left(s_{g}\right)$$

The decoder takes $\Phi \left(s_{t},s_{g}\right)$ as the input and outputs the reconstructed target image ${\bar{s}}_{g}$. The following Equation (8) defines the overall loss of attention-based image reconstruction.

(8) $$L_{att-recons}={\left\|{\bar{s}}_{g}-s_{g}\right\|}_{2^{*}}^{2}+{\left\|{\bar{s}}_{g}{}^{auto}-s_{g}\right\|}_{2}^{2}+\lambda_{m}{\left\|\psi \left(s_{g}\right)\right\|}_{1}$$

${\left\|{\bar{s}}_{g}-s_{g}\right\|}_{2^{*}}^{2}$ is a parametric number whose specific value is the sum of the squares ${\bar{s}}_{g}-s_{g}$. ${\left\|{\bar{s}}_{g}{}^{auto}-s_{g}\right\|}_{2}^{2}$ is the original autoencoder loss to adjust the feature space. $\lambda_{m}{\left\|\psi \left(s_{g}\right)\right\|}_{1}$ is a penalty term to minimize the locations identified as regions of interest. $\lambda_{m}$ is a hyperparameter that is used to balance the total number of regions of interest. Since there is a penalty on the positions identified as regions of interest, the loss will force the model to select relatively more critical parts from the target image and ignore the background in the observed image to reduce the penalty, as shown in Figure 3.

### 4.4. LSTM-Based Dynamics Network Structure

Consider the states $s_{t}$, $s_{t+1}$, which are encoded as feature vectors $\varphi \left(s_{t}\right)$, $\varphi \left(s_{t+1}\right)$. The inverse dynamics model takes $\varphi \left(s_{t}\right)$, $\varphi \left(s_{t+1}\right)$ as the input and predicts the action ${\bar{a}}_{t}$ of the agent when it transitions from $s_{t}$ to $s_{t+1}$. The forward dynamics model takes $\varphi \left(s_{t}\right)$ and $a_{t}$ as inputs and predicts the next state ${\bar{s}}_{t+1}$ of the agent. This is shown in Equations (9) and (10):

(9) $${\bar{a}}_{t}=g\left(\varphi \left(s_{t}\right),\varphi \left(s_{t+1}\right);\theta_{I}\right)$$

(10) $${\bar{s}}_{t+1}=F\left(\varphi \left(s_{t}\right),a_{t};\theta_{F}\right)$$

where ${\bar{a}}_{t}$ is the predicted action and $\theta_{I}$ is the network parameter of the inverse dynamics model, which is trained and optimized by $\underset{\theta_{I}}{\min}L_{I}\left({\bar{a}}_{t},a_{t}\right)$. ${\bar{s}}_{t+1}$ is the predicted state and $\theta_{F}$ is the network parameter of the forward dynamics model, which is trained and optimized by $\underset{\theta_{F}}{\min}L_{F}\left({\bar{s}}_{t+1},s_{t+1}\right)$.

LSTM is capable of modeling sequences and capturing long-term dependencies within input sequences. Integrating LSTM with dynamic auxiliary tasks allows the model to capture intricate patterns and structures in the input sequence, leading to a more robust feature representation. In this study, we adopt a residual network to fit the forward dynamics model. This choice is motivated by the observation representation’s relatively large dimension and the actions’ discrete nature. Utilizing residual networks allows for the better preservation of the action information during the computation of the forward model, resulting in an improved representation of the environment.

**Figure 4:**
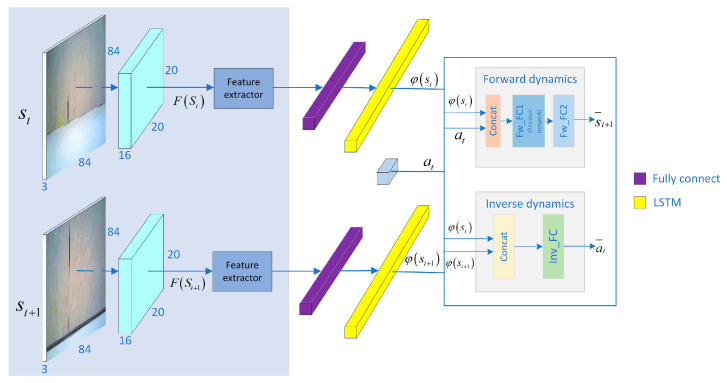
LSTM-based dynamic network structure.

The network inputs are adjacent observed images, passing each image into a separate stream consisting of two convolutional layers with shared-weight parameters. The first convolutional layer is shared with RL, followed by a fully connected linear layer, which is then passed to the LSTM layer to obtain the feature vectors $\varphi \left(s_{t}\right)$ and $\varphi \left(s_{t+1}\right)$. The feature vectors combined with the action $a_{t}$ are input to the dynamics module to obtain the predicted action ${\bar{a}}_{t}$ and the predicted state ${\bar{s}}_{t+1}$, as shown in Figure 4:

### 4.5. Additional Auxiliary Tasks

#### 4.5.1. Pixel-Control Model

The pixel-control auxiliary task defines an additional pseudo-reward function to maximize the absolute pixel change. The additional pseudo-reward function is the pixel-level difference between the current and next states. This additional pseudo-reward function is usually combined with the task’s reward to achieve better training performance. Similar to [18], in the navigation task, this paper uses the pixel-level difference between observations as an additional pseudo-reward based on the pixel-level difference between observations. Specifically, the input observations are initially cropped. Then, the absolute values of the pixel differences between neighboring observations are calculated and pooled equally to the size of each cell. Finally, this paper averages the pooled values over each time step to obtain the pseudo-reward. We compared the pseudo-reward with the action value and calculated the mean square error (MSE) between them as a loss function for the pixel control auxiliary task.

The input to the pixel-control network is the target image and the observed image. The structure of the feature-extraction module is the same as that of the PAAC algorithm. The output of the LSTM in the feature extraction module is connected to the pixel-control module, which consists mainly of a deconvolution layer that up-samples the low-dimensional features back to the size of the down-sampled observations. For each action, the last layer outputs an estimate of the action value for each pixel.

#### 4.5.2. Reward Prediction Model

The reward prediction auxiliary task provides additional learning signals by predicting external reward signals in the environment, thus helping the agent better learn the navigation task. In navigation tasks, reward signals in the environment may be sparse or only present when the task is completed, making it difficult for agents to learn information about how to act from the reward signals. Using reward prediction as an auxiliary task can help the agent learn more about the reward signals, thus improving its learning efficiency and performance.

Referring to [18], in this paper, the agent predicted the next reward based on the past three observations. The input to the reward prediction network was the target image and the observed image. The convolutional layer part of the feature extraction module has the same structure as the PAAC algorithm. The output of the convolutional layer part goes through the fully connected layer, and the final fully connected layer outputs the predicted reward case (positive, negative, or zero).

#### 4.5.3. Depth Prediction Model

The input of the depth prediction model was the target image and the observed image. The convolutional layer part of the feature extraction module has the same structure as the PAAC algorithm, and the output of the convolutional layer part is spanned by a fully connected layer and then reconstructed by decoding the features through a deconvolutional neural network. The loss function for depth prediction is usually calculated using the mean-squared error, and the sum of squares of the pixel-by-pixel difference between the predicted depth image and the actual depth image was found to be the mean value.

## 5. Experiment

The navigation performance of our method was evaluated using simulation environments and real-world image datasets. We demonstrated the improvement in training efficiency by incorporating an LSTM-based dynamics model and attention-based image reconstruction model as auxiliary tasks through ablation experiments.

### 5.1. Implementation Details

The optimizer uses RMSprop, and the training uses a workstation with NVIDIA GTX 3060 GPU with 12 GB of VRAM, an Intel Core processor i5-12600KF (3.90 GHz × 12), and 16 GB of DDR4 RAM. The detailed training parameters are shown in Table 1, where f denotes the number of frames processed so far.

### 5.2. Evaluation Metrics and Baseline Algorithms

#### 5.2.1. Evaluation Metrics

The evaluation metrics in this paper include the navigation success rate, and the mean traveled distance and the mean number of simulation steps.

The success rate (SR): The proportion of the number of times an agent successfully arrives at a specified target location within a specified time step to the total number of tasks. The navigation success rate is one of the critical metrics for evaluating the performance of a visual navigation model because it directly reflects the ability of the model to complete the navigation task. A higher success rate usually indicates a better performance.

The mean traveled distance (MT): The distance traveled by the agent from the starting position to the target position. For the same navigation task, if the model has better navigation performance, then the path length it travels should be shorter, and therefore its mean traveled distance should be smaller.

The mean number of simulation steps (MS): The number of simulation steps (i.e., mean episode length) is usually the number of steps required for the model to make inferences and decisions. Fewer simulation steps usually indicate a more efficient model. Since the time and computational resources required for model inference and decision-making are usually large, reducing the mean number of simulation steps can reduce the time and computational cost of the navigation task, thus improving the efficiency of the navigation model.

#### 5.2.2. Baseline Algorithms

In this paper, the performance of the visual navigation was compared with the following models:

PAAC [39]: PAAC is a parallel advantage actor-critic that can achieve the navigation task only using a visual input.

UNREAL [30]: UNREAL uses unreal self-supervised auxiliary tasks for target-driven visual navigation.

A2C-VN [19]: A2C-VN uses the PAAC algorithm, off-policy critic updates, and the image reconstruction auxiliary tasks to help the agent directly learn the navigation strategy from the input of the original high-dimensional image.

### 5.3. Simulated Environment Experiments

Dmhouse simulator: Kulhánek [19] developed the Dmhouse simulator based on the Quake III Arena and DeepMind Lab. In the real world, the trial-and-error process of reinforcement learning agents may result in the agent getting hurt or damaging the environment. In the simulator, the agent can try various actions in a safe virtual environment and continuously learn and optimize its strategies, avoiding potential risks in the real world. The Dmhouse simulator allows agents to move and collect objects in a synthetic environment and produce highly variable and realistic simulated environments.

**Figure 5:**
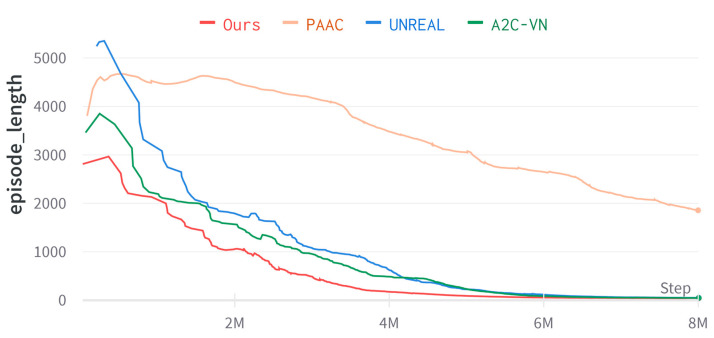
Learning curve of episode length in Dmhouse simulator.

In this paper, the navigation performance of the proposed method was evaluated in an environment generated by a Dmhouse simulator and compared with related baseline algorithms. We randomly sampled the starting position and the target. The algorithm had trained $8\times 10^{6}$ frames, and the training performance is shown in Figure 5. The method of this paper is compared with A2C-VN [19], PAAC [39], and UNREAL [30].

The evaluation was performed in a 100 randomly generated environment with randomly selected starting positions and targets for 1000 simulations, and the results are shown in the following Table 2.

Results and analysis: As seen in Figure 5, our method reaches convergence at about 4 million time steps, the A2C-VN and UNREAL algorithms converge at about 6 million time steps, and the PAAC algorithm does not converge at 8 million time steps. Therefore, our method significantly improves the training efficiency of the model. Table 2 summarizes the test results. It is difficult for the conventional PAAC algorithm to perform well in the highly variable and realistic simulation environment generated by Dmhouse. Regarding the navigation success rate, our method achieves similar results to A2C-VN and UNREAL. However, our method is better regarding mean simulation steps and mean navigation distance.

### 5.4. Real-World Datasets Experiments

Real-world datasets: [19] used the turtlebot2 robot with an RGB-D camera to collect image datasets with 0.2 m resolution in an office environment. In the experiments with real-world image datasets, the initial position is sampled uniformly from the initial position set, and the initial direction is chosen randomly. The robot is judged to have reached the target position when the Euclidean distance between the robot and the target position is at most 0.3 m, and the angle between the robot direction and the target direction is the same. We use the mean goal distance (GD) as a metric to evaluate the robot’s ability to stop close to the target position, as in [19].

In this paper, the navigation performance of the proposed method was evaluated in the context of real-world datasets and compared with related baseline algorithms. We randomly sampled the starting position and the target. The algorithm trained for $1\times 10^{7}$ frames, and we conducted the training in a curriculum-learning method. That is, the complexity of the environment was gradually increased during the training process. Adjusting the initial set of positions achieved the complexity of the environment. To make the task easier for the agent, we first sampled the initial states closer to the target and gradually increased the distance between the initial state and the target. Assuming τ∈[0, 1] to be the environment complexity, we defined the maximal sampling distance ${\mathrm{dmax}}_{E}\left(\tau \right)$ of an environment E as follows:

(11) $${\mathrm{dmax}}_{E}\left(\tau \right)=\tau \underset{s_{1},s_{2}}{\max}\left\{dist\left(s_{1},s_{2}\right)\right\}$$

where $dist\left(s_{1},s_{2}\right)$ measures the distance between any two states of the given environment E. We used Euclidean distance to measure the distance between the corresponding agent positions in the environment.

The initial state was sampled from the uniform probability distribution over the set of possible initial states closer to any target than ${\mathrm{dmax}}_{E}\left(\tau \right)$:

(12) $$\cup \left(\left\{s_{1}|s_{1}\in S_{s},s_{2}\in S_{t},dist(s_{1},s_{2})\leq {\mathrm{dmax}}_{E}\left(\tau \right)\right\}\right)$$

where the set of target states is denoted by $S_{t}$. The environment complexity *τ* starts at 0.3 and gradually increases during the training to 1.0.

**Figure 6:**
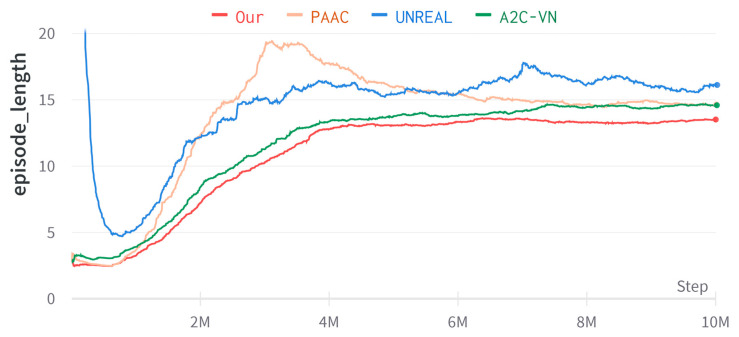
Learning curve of episode length in real-world datasets.

The training performance is shown in Figure 6. The method of this paper is compared with A2C-VN [19], PAAC [39], and UNREAL [30].

The starting position and target were randomly selected. The results of the average number of simulated steps, the average navigation success rate, and the average navigation distance calculated over 1000 simulations are shown in the following Table 3:

Results and analysis: As seen in Figure 6, the initial environment complexity is three steps, and the environment complexity is gradually increased to reach the maximum environment complexity at 4.5 million time steps. The average episode length of our method is always the shortest in comparison, so our method has the best decision-making capability. The shortest path is from the initial to the goal location. Here, it is considered the ideal result. As seen from Table 3, compared with the comparison algorithms, our method performs best regarding average navigation success rate, average simulated steps, and average navigation distance.

**Figure 7:**
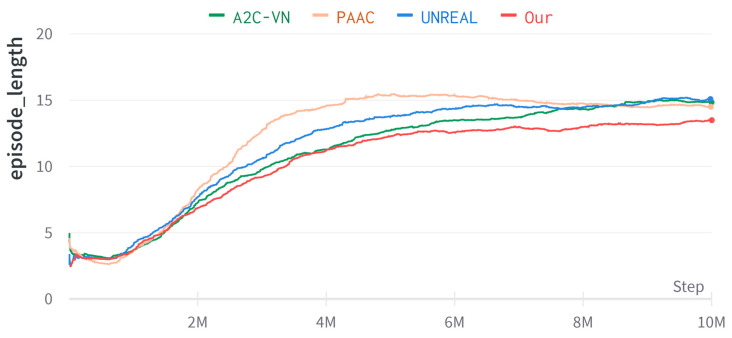
Learning curve of episode length in real-world datasets.

We also evaluated the navigation performance of the proposed method in this paper in the context of real-world datasets using features trained in Dmhouse as pre-trained features. The same as before, the algorithm trained for $1\times 10^{7}\;$frames, and we conducted the training in curriculum-learning method. We randomly sampled the starting position and the target. The training performance is shown in Figure 7. The method of this paper is compared with A2C-VN [19], PAAC [39], and UNREAL [30].

The starting position and target were randomly selected. The results of the average number of simulated steps, the average navigation success rate, and the average navigation distance calculated over 1000 simulations are shown in the following Table 4:

Results and analysis: As seen from Table 4, compared with the comparison algorithms, our method had the best performance in terms of average navigation success rate, the average number of simulated steps, and the average traveled distance. Table 4, compared with Table 3, shows that the algorithm in this paper and A2C-VN improve the navigation performance by using pre-trained features, and UNREAL and PAAC methods reduce the navigation effect after using pre-trained features instead. The navigation effect is reduced by using pre-trained features. The shortest path is about 12.595 steps, and our method is about 13.762 steps, the closest to the ideal result.

### 5.5. Ablation Experiments

This subsection performs ablation of our method to understand the results further. “Ours” is the multi-auxiliary tasks visual-navigation algorithm proposed in this paper, “Ours w/o Att” is the attention-based image-reconstruction model removed, and “Ours w/o Att and Dyn” is the attention-based image-reconstruction model and the LSTM-based dynamics model removed. The algorithm was trained for $8\times 10^{6}$ frames in the environment generated by the Dmhouse simulator.

**Figure 8:**
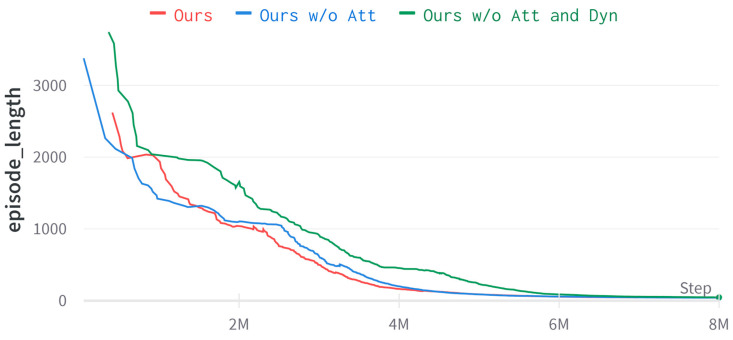
Learning curve of episode length in Dmhouse simulator.

**Figure 9:**
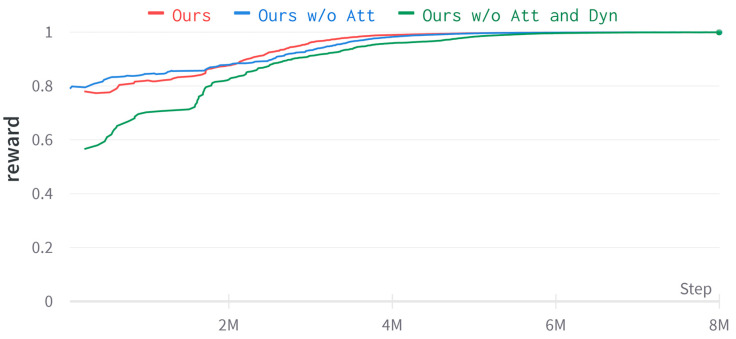
Learning curve of reward in Dmhouse simulator.

As seen in Figure 8 and Figure 9, “Ours w/o Att and Dyn” learns a stable navigation strategy at approximately 6 million time steps, “Ours w/o Att” learns a stable navigation strategy at approximately 4 million time steps, and “Ours” learns a stable navigation strategy at approximately 3.5 million time steps, which shows that our method learns faster. As seen from Table 2, the average number of simulated steps and the average traveled distance can be improved by adding an LSTM-based dynamics model. Adding the attention-based image reconstruction model achieves the highest navigation success rate.

## 6. Conclusions

This paper proposes multiple self-supervised auxiliary tasks for DRL-based target-driven visual navigation. The LSTM-based dynamics auxiliary task utilizes the LSTM network to help the dynamics model better capture the complex patterns and structures in the input sequence, thus providing a more robust feature representation for the navigation task. The attention-based image reconstruction auxiliary task uses attention networks to reconstruct the target image from the observed image. By introducing the attention networks, the model can pay more attention to the critical regions in the navigation task while generating the scene map, making it easier for the navigation agent to understand the scene and thus make more accurate decisions. Experimental results on the simulator and real-world image datasets show that the method proposed in this paper outperforms the robust baseline algorithms.

Further research can be conducted based on this paper. Since the usefulness of auxiliary tasks may change during the learning process, the adaptive adjustment of the weights of multiple self-supervised auxiliary tasks can be considered so that the auxiliary tasks can help the performance of the navigation task the most. We can also consider deploying our model on a real robot.

## Figures and Tables

**Table 1:** Parameters of training.

Parameter Name	Parameter Value
Replay buffer size	2000
Discount factor	0.99
Learning rate	$\max\left(0.7\times 10^{-4}\left(1-\frac{f}{4\;\times \;10^{7}}\right)\right)$
Number environment	16
Maximum rollout length	20 steps
Value replay weight	1.0
Pixel control weight	0.8
Reward prediction weight	1.0
Depth-map prediction weight	0.02
Image reconstruction weight	0.01
Forward dynamics weight	0.5
Inverse dynamics weight	0.5
Pixel control discount factor	4
Pixel control downsize factor	4
Actor weight	1.0
Critic weight	0.5
Entropy gradient weight	0.001

**Table 2:** Comparison of navigation performance of all compared methods in simulated environment.

Method	SR	MT (m)	MS
A2C-VN	0.999	7.796	42.652
UNREAL	0.998	8.612	49.714
PAAC	0.434	66.913	396.414
Ours	1.000	7.089	37.259
Ours w/o Att	1.000	7.209	39.040
Ours w/o Att and Dyn	0.999	7.598	41.950

**Table 3:** Comparison of navigation performance of all compared methods in real-world datasets.

Method	SR	GD (m)	MS
**Our**	**0.878**	**0.184**	**14.966**
A2C-VN	0.857	0.203	15.467
UNREAL	0.810	0.242	16.526
PAAC	0.842	0.233	16.854
Shortest path	-	0.073	12.595
Random	0.205	1.467	147.956

**Table 4:** Comparison of navigation performance of all compared methods in real-world datasets.

Method	SR	GD (m)	MS
**Our**	**0.905**	**0.155**	**13.762**
A2C-VN	0.879	0.184	15.229
UNREAL	0.636	0.535	17.637
PAAC	0.804	0.267	15.317
Shortest path	-	0.073	12.595
Random	0.205	1.467	147.956

## Data Availability

Not applicable.
